# How about the quality and recommendation on prevention, diagnosis, and treatment of HIV/AIDS guidelines developed by WHO: A protocol for systematic review

**DOI:** 10.1097/MD.0000000000023638

**Published:** 2020-12-24

**Authors:** Qingshuang Zhu, Pengzhong Fang, Yadong Zhao, Dingmei Dai, Xiaofeng Luo

**Affiliations:** a School of Public Health; b The First Clinical Medical College, Lanzhou University; c Sexually Transmitted Disease and Acquired Immune Deficiency Syndrome Prevention Branch, Gansu Provincial Center for Disease Control and Prevention, Lanzhou City, Lanzhou, Gansu Province, China.

**Keywords:** AGREE II, guidelines, HIV/AIDS, quality, RIGHT, world health organization

## Abstract

**Background:**

Human Immunodeficiency Virus/Acquired Immune Deficiency Syndrome (HIV/AIDS) has become a pandemic that has infected millions of people around the world and brings a tremendous economic burden. There are numerous guidelines for prevention, diagnosis, and treatment of HIV/AIDS published in recent years, but the quality of these guidelines is still unknown. Therefore, we conducted this study to evaluate the quality of prevention, diagnosis, and treatment of HIV/AIDS published by World Health Organization (WHO) as well as perform a comparison of recommendations between those guidelines. And we also hope our finding could provide suggestions to enhance the quality of future guidelines in this area.

**Methods:**

We obtained guidelines from WHO Guidelines approved by the Guidelines Review Committee (GRC). Two reviewers will independently select eligible guidelines. The quality of included guidelines will be appraised by at least four reviewers through AGREE II and RIGHT tools. The results will be checked for discrepancies. Differences between them than two reviewers will be considered as discrepant and the final discrepancies will be resolved by consensus. The results will be presented in tables and the descriptive statistics will be calculated for all domains of the AGREE II instrument as standard score and median (range) as the reporting quality result of eligible guidelines will also be evaluated through RIGHT criteria. In this study, we will also compare the differences and similarities of recommendations among different guidelines.

**Results:**

The results of this study will increase the knowledge about the development of recommendations guidelines for HIV/AIDS of high methodological rigor and reporting quality. This study may also identify potential limitations for future research in this area.

**Conclusion:**

This study may guide health professionals, policy makers, and health policy managers in choosing the guidelines for recommendation to better to achieve the 90–90–90 targets.

**INPLASY registration number:**

INPLASY2020110010

## 1 Introduction

The prevalence of Human immunodeficiency virus/Acquired Immune Deficiency Syndrome (HIV/AIDS) varies among countries,^[1]^ especially in the low-income and middle-income countries has remained a big challenge to the public health authorities.^[2,3]^ According to the data from the UNAIDS (the Joint United Nations Programme on AIDS), 38 million people were living with HIV/AIDS, 690 thousand AIDS-related deaths and 1.7 million new infections around the world in 2019. As well the government and society spend a lot on the prevention and treatment of HIV/AIDS, which brings a tremendous economic burden.^[2]^

With the advent of antiretroviral therapy (ART) has reshaped the HIV epidemic, changing HIV disease from a fatal condition to a manageable, chronic disease.^[4]^ In developing or developed county, life expectancy for people living with HIV/AIDS (PLWHA) has increase near to the general population.^[1,4–6]^ However, PLWHA experience high levels of a host of physical,^[7]^ cognitive,^[8]^ and emotional^[9]^ comorbidities than the general population. All of those risk factors have a significant impact on PLWHA may led to a decline in their adherence to ART and result in poor treatment outcomes such as drug resistance. Those exposures may increase the risk for worse functioning, disability, poor health outcomes, and low quality of life.

There are so many recommendations for HIV/AIDS guidelines about prevention, diagnosis, and treatment could be found, which produced by different countries and professional organizations. This variation may have been attributed to such factors: cultures, religions, social and economic level, incidence, and the prevalence are varying between different regions and countries. And most of them were focused on high HIV prevalence, resource-limited settings than other settings.

Guidelines play an important role in the prevention, diagnosis, and treatment of HIV/AIDS. For example, so many studies indicate that male circumcision as a preventive intervention can reduces a man's risk of heterosexual infection of HIV about 60%.^[10–12]^ The quality of published guidelines was reported high variability. High-quality guidelines can standardize clinicians’ treatment behaviors, reduce the costs and improve the quality of healthcare. Between them some guidelines are lack of specificity for the implementation of recommendations and lack of stakeholder participant throughout the guideline development process. The guideline developed by the World Health Organization (WHO) should according to the WHO standards and requirements during the development process. To meet the WHO standards, high quality guidelines must take acceptable and feasible into account, the development process contained internal and external consultation with experts, national programme managers, consumer advocates, and evidence review methodologist should also be involved. With the elaboration of these documents, the concerns related to their quality increased. But, to the best of our knowledge, we do not found any systematic review of HIV/AIDS guidelines developed by WHO, at the same time, we do not know that how about the quality of those guidelines. This study will appraise the rigor of the development of WHO guidelines and identify potential limitations in current WHO guidelines for prevention, diagnosis, and treatment of HIV/AIDS.

## 2 Methods

### 2.1 Study design

This systematic review of WHO guidelines for prevention, diagnosis, and treatment of HIV/AIDS will be carried out to assess the quality of WHO published guidelines using both the Appraisal of Guidelines for Research and Evaluation II (AGREE II) and Reporting Items for Practice Guidelines in healthcare (RIGHT) tools to evaluate the quality of those guidelines.

### 2.2 Protocol and registration

This systematic review will be registered in the International Platform of Registered Systematic Review and Meta-analysis Protocols (INPLASY) database November 2, 2020 (protocol number: INPLASY2020110010). Ethical approval is not necessary because we only extract published data or information from published guidelines. If protocol amendments are made, we will record and elaborate on the rationale and details of such amendments in the final. This protocol will be strictly reported complies with Preferred Reporting Items for Systematic Reviews and Meta-Analyses (PRISMA-P).

### 2.3 Eligibility criteria

#### 2.3.1 Inclusion criteria

**Figure 1:**
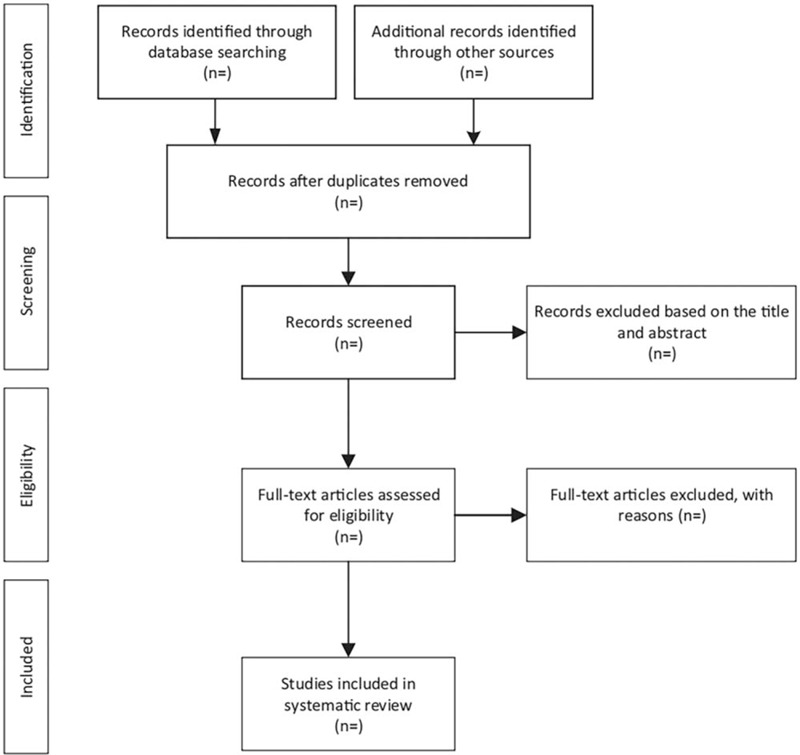
The process of study selection.

We will only include guidance documents that contain guidelines, recommendations, policy statements and manuals about prevention, diagnosis, and treatment of HIV/AIDS written in English and published by WHO. If there is another more up-to-date version of a guideline, we will only add the latest version into our study. Literature screening flow chart is shown in Figure 1. Besides, this study not only includes guidelines, but also contains other supporting information. We will describe it in detail below.

#### 2.3.2 Exclusion criteria

WHO published guidelines about tuberculosis and/or hepatitis B/C and mentioned prevention, diagnosis, and treatment of HIV/AIDS. The guideline is incomplete or only contains a summary; the guideline is not in English; a guideline is clearly a duplicate publication to another selected guideline.

#### 2.3.3 Measured outcome

The methodological quality of eligible guidelines will be evaluated, and the score of every domain will be identified by using the AGREE II tool. The purpose of developing AGREE II was creating a way to assess the methodological quality of guidelines in common and transparent manner,^[13]^ which has been widely used.^[14,15]^ However, AGREE II was created by a small group of researchers, and did not separate out reporting and methodological quality of guidelines. Using it we cannot clear that the exact quality of the guidelines.

In 2017, the International Reporting Items for Practice Guidelines in Heath Care (RIGHT) group working group developed the RIGHT checklist to help guideline developer in reporting guidelines.^[14]^ Because the different purpose, structure, and content,^[16]^ so we will also evaluate reporting quality of guidelines through the RIGHT checklist, because RIGHT is better than AGREE II when it is used to assess the reporting quality of guidelines and it has been widely identified as the reporting standard for guidelines.^[14]^

### 2.4 Search strategy

This study combines the authority of information sources, we obtained guidelines from a WHO Guidelines approved by the Guidelines Review Committee (GRC), which aimed to ensure that WHO guidelines are of high methodological quality and are developed through a transparent, evidence-based decision-making process. So, we will include all eligible documents from the WHO Guidelines approved by the Guidelines Review Committee HIV/AIDS topic (https://www.who.int/publications/guidelines/hiv_aids/en/).

Last but not least, we will also hand search the WHO website for available eligible guidelines, recommendations, and policy statement that are not found before. If necessary, we will contact with authors and consult leading experts in this field of HIV/AIDS to avoid where important information is missing.

### 2.5 Study selection and data extraction

#### 2.5.1 Determination of eligibility of the study

Two reviewers (QSZ, PZF) will independently read the titles and abstracts of all searched guidelines and determine whether they meet the eligibility criteria. For meet the inclusion criteria, titles and abstracts may be guideline or recommendations or document written in English and published by WHO relevant to prevention, diagnosis and treatment of HIV/AIDS. Titles and abstracts should be recognized by at least one reviewer of them, full texts will be reviewed in the same way by two reviewers (YDZ, DMD) using the same eligibility criteria. We will remove duplicates by one of the reviewers (QSZ). Discrepancies will be solved through discussion until the consensus be achieved. If necessary, we will involve the third reviewer (XFL) to help reach the final decision.

#### 2.5.2 Data extraction

All reviewers will complete training of AGREE II and RIGHT tools until reviewers can competence this work. We will use a standardized and pilot-tested data extraction form to extract following data: year of publication, type of guidelines (first edition or updated or revised), type (diagnosis, prevention and control, therapeutic, pre-exposure prophylaxis (PrEP) or post-exposure prophylaxis (PEP), and/or others), funding source, methodologist, update plan of a guideline, update cycle of a guideline, peer-review, presentation literature search strategy or not, presentation literature inclusion and exclusion criteria or not, how to classifying the quality of evidence (such as Grading of Recommendations Assessment, Development and Evaluation (GRADE) or others), methods of reach consensus (iterative discussion, the Delphi approach, not mentioned or Nominal Group Technique), guideline development methods (GRADE or others).

### 2.6 Quality assessment of guidelines

The quality of all selected guidelines will be appraised through AGREE II and RIGHT tools. AGREE II is an international, validated and rigorously developed tool to evaluate the quality of guidelines and consensus statement.^[15]^ AGREE II contains 6 quality domains consists of 23 items, scored with a Likert scale of 1 (totally disagree) to 7 (totally agree) for every reviewer to each item.^[17]^ The six domains are: scope and purpose, stakeholder, and involvement, rigor of development, clarity of presentation, applicability, editorial independence.

Due to it is not clear that the exact quality of the guidelines, so we will use RIGHT instrument to evaluate the reporting quality of guidelines. And the RIGHT checklist includes 22 core items that are considered necessary for good reporting of guidelines: basic information (items 1–4), background (items 5–9), evidence (items 10–12), recommendations (items 13–15), review and quality assurance (items 16–17), funding and declaration and management of interests (items 18–19), other information (items 20–22).^[18]^ Reviewers will independently appraise the consistency of guidelines with the RIGHT checklist, and then each item will be categorized as “yes (Y),” “no (N),” “partial (P),” “not applied (NA)” according to the guidelines own reporting standards.

At least four independent reviewers who will be trained to appraise guidelines through using the AGREE II and RIGHT tools performed an independent review of the quality of all selected guidelines. The final decision will be made by consensus if not, another reviewer will be involved to assist them to make the final decision. If necessary, mind mapping will be piloted to presentation the comparison of recommendations.

### 2.7 Data synthesis

We will use the intraclass correlation coefficients (ICCs) to appraise the reliability of measurements or rating. Two ways, mixed effects model was used to calculate reviewers, reviewer average per domain and general with rating consistency. The null hypothesis was the ICC equivalent to 0. When *P* value < .05 indicate the ICC was significantly different from 0. And the ICC value between 0.01 and 0.20 deemed as poor reliability, between 0.21 and 0.40 deemed as fair reliability, between 0.41 and 0.60 deemed as moderate reliability, between 0.61 and 0.80 deemed as good reliability, between 0.81 and 1.00 deemed as excellent reliability.^[19]^ We use SPSS 26.0 software to perform the statistical analysis. We will count the standardization deviation and median of standardization percentage. Meanwhile, the reported percentage of each item in the RIGHT checklist of all CPGs and the reported number and percentage of all items in each CPGs will be performed the statistical analysis.

## 3 Discussion

In 2013, WHO set 90-90-90 targets: an ambitious target to end HIV by 2030. The 90–90–90 targets include 90% of the people living with HIV know their HIV status, 90% of the people who know their HIV status receiving ART and 90% of the people receiving ART having suppressed viral loads.^[20]^ High quality guidelines can help us to reach the targets as soon as possible.

To the best of our knowledge, this study will be the first to be conducted to appraise the quality and compare the recommendation among different guidelines regarding prevention, diagnosis, and treatment of HIV/AIDS published by WHO. This systematic review will select guidelines of high-quality, we hope this study provide a synthesis of best practice for health-care workers, policy and decision-makers, programme manager, health-care provider and implementing agencies, which we anticipate will inform guidelines and practice standards. Our finding may assist the development of recommendations guidelines for HIV/AIDS of high reporting quality and high methodological rigor, which will improve our opinion on how guideline development methods and processes may affect the similarity or differences of the guidelines. In addition, we hope our study will further address potential limitations in developing guidelines and provide insight and direction for future research.

However, there are several limitations exist in our study. First, we will only include guidelines published by WHO written in English, which will reduce the number of eligible guidelines and limit the coverage of countries and organizations if they also developed relevant guidelines do not in English. Second, despite we will choose both AGREE II and RIGHT instruments measure the outcome and we believe that both of them contain the most comprehensive process for guidelines development. But we know that some guideline development organizations also adopt other tools. For instance, the 2014 WHO Hand book for Guideline Development^[21]^ and the Institute of Medicine standards^[22]^ are also frequently used to develop high quality guidelines. Thirdly, we will only identify all guidelines that submitted to WHO GRC, so important documents which meet our standards but not submitted to WHO GRC in time may be missing.

We believe that we will make recommendations with the explicit inclusion criteria, extensive and comprehensive research of eligible guidelines, independent appraisal of eligible guidelines. Meanwhile, we believe we will finally achieve the last of the WHO ambitious 90-90-90 targets with unremitting efforts.

## Acknowledgments

We thank Dr Jinhui Tian for his guidance on the methodology of this study.

## Author contributions

QSZ, PZF, YDZ, and DMD searched for the literature. QSZ, PZF, and XFL conceived this study, designed the inclusion/exclusion criteria and the searching strategy. QSZ and XFL drafted the protocol and revised the manuscript. YDZ and DMD designed a data extraction table, collected the data. YDZ will make statistical analysis. All authors approved the final version of the manuscript.

**Conceptualization:** Qingshuang Zhu, Pengzhong Fang, Xiaofeng Luo.

**Data curation:** Qingshuang Zhu, Pengzhong Fang, Yadong Zhao, Dingmei Dai.

**Funding acquisition:** Xiaofeng Luo.

**Investigation:** Qingshuang Zhu, Pengzhong Fang.

**Methodology:** Xiaofeng Luo.

**Software:** Yadong Zhao.

**Writing – original draft:** Qingshuang Zhu, Xiaofeng Luo.

**Writing – review & editing:** Qingshuang Zhu, Xiaofeng Luo.
